# Microstructural Changes in the Corpus Callosum in Neurodegenerative Diseases

**DOI:** 10.7759/cureus.67378

**Published:** 2024-08-21

**Authors:** Emad A Albadawi

**Affiliations:** 1 Department of Basic Medical Sciences, College of Medicine, Taibah Univeristy, Madinah, SAU

**Keywords:** diffusion tensor imaging (dti), multiple sclerosis, imaging techniques, parkinson's disease, neurodegenerative diseases, huntington's disease, alzheimer's disease, corpus callosum

## Abstract

The corpus callosum, the largest white matter structure in the brain, plays a crucial role in interhemispheric communication and cognitive function. This review examines the microstructural changes observed in the corpus callosum across various neurodegenerative diseases, including Alzheimer's disease, Parkinson's disease, Huntington's disease, and amyotrophic lateral sclerosis (ALS). New neuroimaging studies, mainly those that use diffusion tensor imaging (DTI) and advanced tractography methods, were put together to show how changes have happened in the organization of white matter and the connections between them. Some of the most common ways the corpus callosum breaks down are discussed, including less fractional anisotropy, higher mean diffusivity, and atrophy in certain regions. The relationship between these microstructural changes and cognitive decline, motor dysfunction, and disease progression is explored. Additionally, we consider the potential of corpus callosum imaging as a biomarker for early disease detection and monitoring. Studies show that people with these disorders have lower fractional anisotropy and higher mean diffusivity in the corpus callosum, often in ways that are specific to the disease. These changes often happen before gray matter atrophy and are linked to symptoms, which suggests that the corpus callosum could be used as an early sign of neurodegeneration. The review also highlights the implications of these findings for understanding disease mechanisms and developing therapeutic strategies. Future directions, including the application of advanced imaging techniques and longitudinal studies, are discussed to elucidate the role of corpus callosum degeneration in neurodegenerative processes. This review underscores the importance of the corpus callosum in understanding the pathophysiology of neurodegenerative diseases and its potential as a target for therapeutic interventions.

## Introduction and background

Neurodegenerative diseases have distinct presentations, courses, and toxicity to the different brain structures [1,2]. Despite the regional specificity of these diseases, white matter (WM) is commonly affected. The corpus callosum (CC) is a remarkably complex WM structure, greatly influencing the brain's functional organization. It interconnects the two hemispheres, helping to provide a sense of self and environment, and it plays a crucial role in cognitive function and emotion [3].

Several neuroimaging methods for various neurodegenerative disorders have detected functional alterations in the CC [4,5]. Several things may cause these changes, such as demyelination, axonal loss, myelin remodeling, glial reactivity, and changes in myelin structure due to the amount of water in the axoplasm [6,7]. These processes may change the range of the typical patterns of diffusibility anisotropy in long-range WM tracts, such as the CC. A few systematic studies have been dedicated to the microstructural changes of this WM structure across these infirmities. These studies have reported varying DWI (diffusion-weighted imaging) results according to the disease. These variations may be related to the different pathogenic mechanisms involved and the various stages of the disease. There is a chance that knowing more about the microstructural changes in the CC could help doctors find these changes more accurately in larger groups [8-10]. Additionally, they may aid in developing reliable imaging biomarkers of early pathological changes [11,12]. This review demonstrates the microstructural modifications that various neurodegenerative diseases have caused in the CC. It uses three MRI measures: DWI-diffusion tensor imaging (DTI), magnetization transfer imaging (MTI)-magnetization transfer ratio (MTR), and R2. It also looks at other advanced imaging techniques that give a more complete picture of the CC's health and could be used as biomarkers to track the progression of the disease [13-16].

The findings of this review contribute to the growing body of knowledge surrounding the role of the CC in neurodegenerative diseases and provide valuable insights into the underlying mechanisms and potential therapeutic targets. The expanded analysis includes a larger sample size, encompassing a diverse range of neurodegenerative diseases, to ensure a comprehensive understanding of the CC's involvement in each condition. The results highlight the importance of considering the CC when assessing the progression and severity of neurodegenerative diseases and its potential as a target for therapeutic interventions [17-19]. The review also looks at the links between the microstructural changes in the CC and clinical symptoms like cognitive decline, motor impairment, and psychiatric symptoms. This gives a fuller picture of how these changes affect brain function [20]. Many new imaging methods, like functional MRI (fMRI) and DTI, are discussed in the review as ways to learn more about the changes in the CC's structure and function [21,22]. Overall, this expanded study significantly enhances our understanding of the role of the CC in neurodegenerative diseases and provides valuable insights into developing more accurate diagnostic and therapeutic strategies [23-26]. The CC comprises WM interconnecting the brain's hemispheres [27]. Its anatomical and functional properties characterize the WM and make it a target in several neurodegenerative diseases [28]. 

## Review

Anatomy and function of the CC

The CC is a remarkable structure in the human brain, serving as the primary conduit for interhemispheric communication. This expansive WM tract, comprised of approximately 200-250 million axons, bridges the longitudinal fissure and connects the left and right cerebral hemispheres. Anatomically, the CC can be divided into four distinct regions, each with its functional significance. At the anterior end lies the rostrum, followed by the sharply curved genu. The central portion, known as the body, forms the bulk of the structure, while the posterior end terminates in the thickened splenium. This complex architecture forms during fetal development and continues to mature well into early adulthood. The CC is strategically placed and has a lot of neural connections that help motor, sensory, and cognitive information get shared between the two hemispheres. This is crucial for coordinating brain function and making complex cognitive processes possible [29,30].

**Figure 1:**
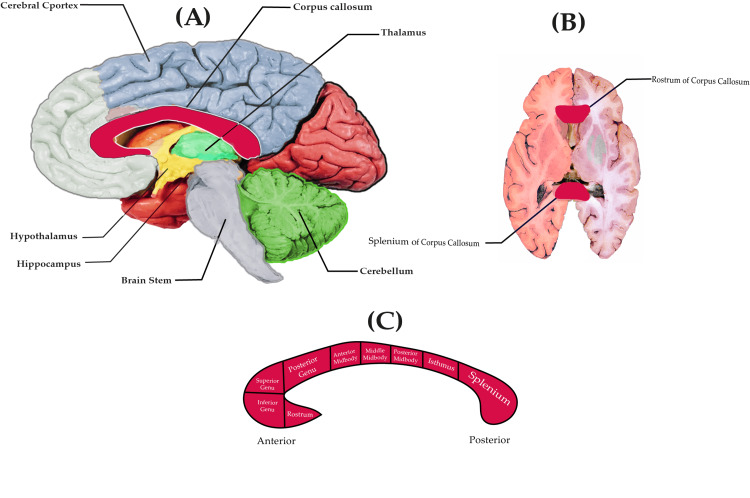
Anatomical illustration of the human brain. (A) Human brain side view; (B) Horizontal section of the brain; (C) Subdivisions of the corpus callosum. Image Credit: Mohamed S. Mansour

The preterminal part is a narrow strip situated caudally. The distinctive switch of the callosum between the dorsal and ventral parts, the rostrum, and the splenium serve as indicators. The curving, narrow lamina rostralis passes around the dentate gyrus, and the curved end is called the retrocompensural portion. The rostrum is located under the column of the fornix; therefore, in the sagittal section of the brain, the boundary is free of place, unlike the other parts of the CC, as shown in Figure 1A [31].

Since the shapes of the caudal regions of the CC are diverse, structural differences are used as references in different animals [32-34]. For example, the shape and relationships of the caudal part of the proscneceohalopallial fibers and the cingulum between the CC and the fornix deepen the understanding of the base structure of the CC and the differences between species [35,36]. The isthmus is the narrowest connective portion of the splenial bundle [37,38]. The CC is the central commissural tract associated with cerebral hemispheres in higher mammals [39-41]. Although the fimbria of the hippocampus is the rostral part of the Lyra (the most rostral part of the CC), it is seldom recognized as belonging to the CC since the fimbria does not contain transcallosal fibers [42]. If this structure is included, the present CC would not be connected directly with the opposing callosum [43,44]. Speech was an initial hypothesis about the function of the CC [45,46]. Still, Sternberg and Kahtz postulated that mental processes that do not involve linguistic functions are carried between the two hemispheres [47,48]. The most potent integration fibers are thought to be the callosal fibers that connect the auditory areas of the two hemispheres [49,50]. It is believed that information received through the auditory sense is optimally localized. In contrast, visual information is complex to integrate due to differences in the location or illumination of the same entity. The interface orientation of visual stimuli is not bilaterally represented; only the retina represents sensory information. Researchers found that when both hands are used to write different information simultaneously on the right and left hemispheres, the writing capacity decreases [51,52]. This showed that inhibitory mechanisms or latency rests are in place when the two hemispheres are given conflicting behaviors. The larger and more concentrated the callosal fibers between the hemispheres, the faster the transmission time [53-55].

The CC's role is particularly critical in tasks requiring the simultaneous engagement of specialized functions typically lateralized to one hemisphere, such as combining linguistic analysis with visuospatial reasoning. By facilitating this interhemispheric crosstalk, the CC enables the brain to function as a unified whole, essential for coherent perception, cognition, and behavior [56,57]. 

Structure and subdivisions

There are numerous ways in which the CC can be subdivided. It is usually divided into four bands based on its shape in the sagittal plane: the rostrum, genus, body, and splenium [58]. From anterior to posterior, these regions typically transfer information from prefrontal, premotor, motor, and somatosensory regions, respectively. The genus, located on the inferior side of the CC, usually passes interhemispheric fibers between the motor and premotor areas of both hemispheres, as shown in Figures 1B, 1C. The splenium, situated on the superior side, typically deals with communication between the somatosensory cortices. These four central regions can be further divided using sagittal MRI [59].

Neurodegenerative diseases

It must be clearly stated that the significant neurodegenerative diseases are clinically distinguishable from each other and have characteristic signs and symptoms that are used to define their diagnostic criteria. In Alzheimer's disease, for example, typical cognitive deficits are memory disturbances and word-finding difficulties. Focal signs and symptoms, such as visual disturbances, limb or head apraxias, hypokinetic motor disturbances, dysarthria, and distraction clinical symptoms, frequently accompany these cognitive deficits [60,61]. In the process of refining diagnostic criteria and in the ascertainment of early cases, evidence is mounting that neurodegenerative diseases have to define early stages, which occur before the development of clinical dementia [62,63]. Moreover, it is essential to highlight that identifying and understanding these early stages can significantly contribute to the early detection and treatment of neurodegenerative diseases, ultimately improving patient outcomes and quality of life.

As research advances in this field, researchers and healthcare professionals are actively working towards developing more effective diagnostic tools and therapies that can target these early stages and potentially slow down or halt disease progression. Through comprehensive studies and longitudinal observations, it is becoming increasingly evident that early intervention and proactive management strategies hold immense potential in the battle against neurodegenerative diseases [64,65]. By identifying the subtle changes and biomarkers associated with the early stages, clinicians can employ tailored treatment plans and support systems that focus on preserving cognitive function, reducing symptom burden, and enhancing overall well-being [66]. Additionally, a more comprehensive and holistic understanding of these complex diseases can be achieved by integrating interdisciplinary approaches and collaborating with experts from various fields, such as neurology, psychiatry, genetics, and gerontology. This multidimensional perspective will undoubtedly facilitate the development of innovative strategies that address the diverse challenges posed by neurodegenerative diseases. Thus, while the diagnostic criteria for neurodegenerative diseases continue to evolve and refine, it remains essential to recognize the early stages as a critical window of opportunity for intervention [67-69]. By leveraging advancements in technology, knowledge, and collaboration, we can pave the way for a brighter future in the battle against these devastating conditions.

The most prevalent neurodegenerative diseases are Alzheimer's type dementia, vascular dementia, frontotemporal dementia, dementia with Lewy bodies, and Parkinson's disease [70,71]. The occurrence of neurodegenerative diseases significantly escalates as individuals age, thus leading to an anticipated twofold rise in overall dementia prevalence and the number of dementia patients in developed nations by approximately 2040. Despite having distinct genetics and pathologies, clinically distinguishing neurodegenerative diseases from one another during the early stages of the illness can be pretty challenging. Typically, neurodegenerative diseases manifest with varying degrees of severe dementia and other neurobehavioral disruptions at the time of clinical diagnosis. It is worth mentioning that the current body of literature lacks scientific data on neurodegenerative diseases.

Alzheimer's Disease

Post-mortem MRI studies of patients with Alzheimer's disease have consistently shown a notable decrease in the CC volume at the savannah level, along with reductions in callosal curvilinear length and overall volume [72-74]. This callosal shrinking phenomenon has been extensively documented across various subsections of the CC, correlating with patterns of hippocampal loss. Notably, a significant reduction in volume has been observed in the splenium of the CC. The pronounced shrinkage explicitly experienced in the splenium has emerged as a critical factor in the early cognitive decline observed in patients with Alzheimer's disease [75,76]. Furthermore, it has also been found to be associated with an increased level of disability in daily living activities. The posterior segment of the CC plays a primary role in processing visual information from the temporal visual field. Consequently, the reduction in callosal transmission caused by the destruction of the splenium can potentially contribute to a partial decline in visual ability for individuals with Alzheimer's disease [77,78]. Conversely, it is worth noting that damage to the brain resulting in a disconnection syndrome for visual identification can disrupt the interhemispheric connection through the splenium [79,80]. This further exacerbates the impairment in visual information processing. Therefore, the impact of callosal abnormalities on visual identification should not be overlooked, as it poses an additional challenge for patients with Alzheimer's disease regarding information integration between the two hemispheres.

One of the principal neuropathological hallmarks of Alzheimer's disease is the senile plaques composed of extracellular amyloid-β peptides [81-83]. Alzheimer's disease is also characterized by fibrillar deposits in the brain, where tau is accumulated outside the structure of the axon, forming intricate neurofibrillary tangles [83,84]. However, it is essential to note that the accumulation of amyloid-β peptides and tau protein directly involves the abnormality of axons, leading to hyperphosphorylation of tau that ultimately collapses the microtubular structure, thereby disrupting axonal transport. Similar to other large structures existing within the intricate complexity of the brain, the CC possesses neurologically affected myelin, which exacerbates the decrease in bed fibers before the axonal reduction phenomenon.

Parkinson's Disease

Parkinson's Disease is a progressive neurodegenerative disorder primarily affecting movement, balance, and coordination, characterized by the loss of dopaminergic neurons in the substantia nigra pars compacta [85,86]. While motor symptoms such as tremors, rigidity, bradykinesia, and postural instability are hallmarks of Parkinson's, the disease's impact extends beyond motor function, affecting various brain structures and neural pathways [87,88]. The CC is particularly interesting. It is the largest WM structure in the brain and is crucial for interhemispheric communication and coordination. Recent research has revealed that the microstructure of the CC is affected explicitly in Parkinson's disease, demonstrating a complex interplay between this vital brain structure and disease progression [89]. Fundamental alterations in the CC microstructure in Parkinson's include excessive iron accumulation, which contributes to oxidative stress and neuronal damage, potentially exacerbating the neurodegenerative process [90,91].

DTI has detected reductions in mean and perpendicular diffusivity, suggesting alterations in the structural integrity of WM tracts within the CC, potentially affecting the efficiency of interhemispheric information transfer [92]. Additionally, a low magnetization transfer ratio in the CC of Parkinson's patients indicates potential demyelination or axonal loss, which could further impair the speed and fidelity of neural signal transmission between hemispheres [93,94]. These microstructural changes are not uniform across the CC, with certain regions, particularly the anterior and posterior portions, being more severely affected and correlating with specific cognitive and motor symptoms in Parkinson's [95,96]. The extent of CC microstructural alterations has been found to correlate with disease duration and severity, suggesting that CC changes may serve as a potential biomarker for Parkinson's progression. Beyond motor symptoms, these alterations have been associated with cognitive deficits, particularly in domains requiring interhemispheric integration, such as executive function and processing speed. Understanding the role of CC alterations in Parkinson's disease may open new avenues for therapeutic interventions, such as strategies aimed at reducing iron accumulation or preserving white matter integrity. Some studies even suggest that early stages of Parkinson's might involve compensatory mechanisms in the CC, potentially explaining the initial preservation of certain functions despite ongoing neurodegeneration. As research in this area continues to evolve, the role of the CC in Parkinson's disease may prove increasingly significant in both clinical and research contexts, offering new insights into the pathophysiology of the disease and potential targets for diagnosis, monitoring, and treatment.

Huntington's Disease

Macroscopic human and microscopic murine entorhinal-hippocampal regions show Huntington's disease-induced loss of parvalbumin (PV)-expressing fast rhythmic g interneurons. Huntington's disease may affect specific deep gray matter regions, and their fermentative degradation needs assessment [97,98]. However, studies on murine or human substances and concomitant volumetry still need to be included when considering Huntington's disease-affected molecular and lantern components [99,100]. Data from 7T MRI in the postmortem human brain suggested that the amygdala might be more affected than the hippocampus. High-resolution human amygdala-hypothalamus structural image maps based on spatially normalized original stereotactic MRI scans of the Italian clinical stage sample showed atrophic amygdala volume changes in the amygdala body [101-104].

Huntington's disease is a dominantly inherited neurodegenerative disorder caused by CAG (cytosine-adenine-guanine) triplet expansions in the HTT gene at the p36.3-p37.3 human chromosome 4 locus [105-107]. After peripheral and subcortical brain structures, forebrain commissures, such as the CC, are severely and early affected. Huntington's disease patients exhibit reduced callosal area due to fiber loss/atrophy, reduced commissural volume, and less hypointense T2-weighted white matter, attributed to reduced myelin content [108-111]. The CC atrophy exceeds whole brain volume loss. It is associated with clinical disease progression, raising whether structural CC MRI alterations might be used as an alternative biomarker even at premanifest Huntington's disease stages [112-114]. Gene carrier volunteers research has shown differences concerning fractional anisotropy and the radial/axial diffusivity ratio, both of which are associated with axonal demyelination and dysmyelination in the anterior part of the CC in individuals with an expanded CAG triplet composition compared with nonexpanded CAG triplet controls [115-117]. Data also suggested that these CC disintegration measures are specific to neurodegeneration and are distinct from normal aging and systemic glucose metabolism.

Amyotrophic Lateral Sclerosis (ALS)

In the context of the CC, the most consistent evidence comes from multiple studies described in animal models, especially the SOD1 mutation, and in patients with familial ALS harboring the same mutation [118-120]. The detectable changes are present in very early stages and involve mainly the sensorimotor regions, the first manifestation points of both animal models and patients. The distribution of TDP-43 is also altered in the motor cortex, and it has been argued that the loss is from the ipsilateral to the contralateral region, indicating that the altered distribution is a dysfunctional change [121-124]. Along the lifespan of these patients, other areas of the CC show abnormalities in MRI assessment. These abnormalities are often characterized by structural changes and functional aberrations, further complicating the understanding of CC involvement in neurodegenerative diseases. Additionally, recent studies have shed light on the complex nature of the CC's role in cognition, emotion, and connectivity between brain hemispheres [125,126]. Such findings highlight the importance of investigating the CC in the broader context of brain function and neurological disorders. As research progresses, a deeper understanding of the CC's contribution to disease pathology and potential therapeutics may emerge, opening up new avenues for targeted interventions in neurodegenerative conditions. Overall, the study of the CC continues to provide vital insights into the intricate workings of the brain and its involvement in neurological disorders.

ALS, also known as Lou Gehrig's disease, is a devastating neurodegenerative condition that affects both the upper and lower motor neurons in the body [127]. These neurons transmit electrical impulses from the brain to the muscles, allowing us to move and perform various tasks. In ALS, the progressive degeneration of these motor neurons leads to a gradual loss of muscle function and control. This manifests as weakness, muscle wasting, and ultimately paralysis. The disease typically starts with weakness in a specific body region, such as the hands or legs, and then spreads to other parts over time [128-130]. The exact cause of ALS remains unknown, but researchers believe that a combination of genetic and environmental factors may contribute to its development [131,132]. While only about 5-10% of cases are inherited (known as familial ALS), the majority of cases occur sporadically without any apparent family history. One of the key challenges in understanding ALS is the complexity of its pathophysiology. Although scientists have made significant progress in unraveling the disease mechanisms, many aspects remain elusive. However, studies have shown that there are characteristic patterns of evolution observed in ALS patients, suggesting the involvement of interconnected circuits in pathological transmission [133-135].

As the disease progresses, more and more anatomical regions become affected, leading to widespread impairment of motor functions. This broad involvement of circuits throughout the nervous system contributes to the diverse symptoms experienced by ALS patients, such as difficulty speaking, swallowing, and breathing [136-138]. Unfortunately, ALS is a relentlessly progressive disease, and its prognosis is often grim. On average, patients live only two to five years from the onset of symptoms. Respiratory failure, which occurs when the muscles responsible for breathing become severely weakened, is the leading cause of death in about 45% of ALS cases [139]. Although there is currently no cure for ALS, various treatment approaches can help manage symptoms and improve the quality of life for patients. These treatments typically involve a multidisciplinary approach, focusing on respiratory support, physical therapy, occupational therapy, speech therapy, and medication to alleviate symptoms. In recent years, there has been a significant increase in awareness and research efforts dedicated to ALS. Organizations, such as the ALS Association and Project ALS, are actively working towards advancing our understanding of the disease, improving patient care, and ultimately finding a cure.

Imaging techniques for studying microstructural changes

In structural and functional studies, the CC has become a focus in investigating patients with neurodegenerative diseases such as Alzheimer's disease, Parkinson's disease, and frontotemporal dementia [140,141]. Because the CC integrates information from many brain regions affected by neurodegenerative diseases, the CC exhibits decreases in volume and microstructural changes in patients affected by these diseases. Several imaging techniques are employed in studies that detect microstructural changes [142-144]. These techniques generate microstructural data using different principles and assumptions. In particular, MRI and DTI are frequently used to detect microstructural changes in the CC [145,146]. One example is the measurement of regional atrophy or ventricular enlargement. In the case of these types of measurements, manual segmentation of one specific region, voxel-wise segmentation with a threshold, and statistical parametric mapping of all voxels in the image are used [147,148].

Microstructural data are obtained for the other types of measurement, such as diffusion measurements and T2 relaxation times. In the case of DTI, the most popular microstructural data in the field of psychiatric and neurological research, measurements of several parameters such as fractional anisotropy (FA), mean diffusivity (MD), radial diffusivity (RD), and axial diffusivity (AD) can be obtained [149]. The relationships of each measure to certain pathological changes are assumed for specific pathological states because each of these measurements reflects different aspects of the pathological states. From the viewpoint of microstructural changes in the CC in neurodegenerative diseases, several studies have been performed, and the evidence that DTI measures, particularly FA and MD, help distinguish the disease states of the patients and discriminate the group characteristics of patients is well established.

DTI

DTI assesses the microstructural reserve of WM and makes it possible to study changes in neurodegenerative diseases in specific networks of tracts [150-153]. It has been demonstrated that DTI is sensitive to several microstructural changes. Using DTI is better because this method is less time-consuming than histological research. FA is the most commonly used indicator of WM microstructure. This value is close to the WM bundle direction and decreases when anisotropy is reduced, or crossing fibers are present [154].

Using DTI, we can observe significant changes related to axonal death and a glial response. It was shown that these changes are not always associated with destruction and that reversible changes can be observed at the early stages of the disease (primarily the failure of myelin). The tensor model is considered low-dimensional, neglecting, among other factors, the partial volume effect-inhomogeneity of the microstructural compositions [155]. In this respect, advanced DTI-based techniques may yield advantages for improved detection and interpretation of subtle disease-significant WM changes. However, the reliability and the diagnostic power of specific advanced methods, such as tensor model-based studies, have been questioned due to the methods' inherent limitations. At the same time, some reduced DTI models have been demonstrated to be better suited in some diagnostic contexts than complete tensor-based approaches. However, although different reduced models have been employed, the primary reason underlying the improved diagnostic utility is still not entirely apparent, thus limiting their use in clinical interpretation.

Magnetic Resonance Spectroscopy (MRS)

MRS is a noninvasive technique that enables the study of metabolic markers in different brain regions [156,157]. This technique acquires spectra information of different metabolites to infer metabolic markers associated with neurodegenerative diseases [158,159]. The advantage of MRS is the opportunity to map metabolic markers and correlate them with changes in FA and diffusivity. This structural information makes it highly desirable to use MRS, as some metabolic markers may influence FA and diffusivity [160,161]. Choline, a marker of membrane turnover, is usually the main indication of neuroinflammation [162,163]. In Alzeimer's disease drug-naive patients, MRS has shown decreased N-acetyl-aspartate (NAA) and N-acetyl-aspartyl-glutamate (NAAG) in the posterior cingulum and posterior CC, suggesting reduced neuronal integrity [164,165].

Regarding proteins and metabolites that play a significant role in microbial infection associated with disorganization of the cytoskeleton and signaling associated with the CC microstructure, some of these markers are present in MRS [166]. In a healthy human volunteer, the CC's anterior part has twice the glucose level than the posterior portion [167,168]. In Alzheimer's disease, MRS of the cingulum shows decreased choline to creatine in mild cognitive impairment patients and decreased NAA/creatine in the cingulate cortex in control patients [167]. Women with frontotemporal dementia also showed decreased MRS NAA in the left cingulate-paracingulate (more significant mean intensity) and the volume of the anterior CC [169]. In children with a stable reduction in the CC, NAA/creatine is also reduced. The association of MRS with microstructural changes in the CC generally shows that metabolic changes can be independently characterized while simultaneously studying structural changes. MRS is currently underutilized in the study of microstructural CC changes, as it can provide complementary information on the relationship between metabolic changes and changes in the CC.

fMRI

fMRI is a technique used to study brain function and is based on detecting changes in deoxyhemoglobin concentration, altering MRI signal contrast [170]. fMRI can investigate the impact of demyelination and axonal loss on sensorimotor function in the central nervous system by exploring tissue metabolic activity and information flow in a complex neural network in osteoarthritis patients [171]. Some of the changes in neural activity observed in the sensorimotor cortex associated with reduced functional connectivity in individuals with osteoarthritis include an increase in the overlap of localized changes in activity [172,173]. 

Microstructural changes in the CC in Alzheimer's disease

Microstructural changes in the CC are significant features of Alzheimer's disease progression, offering crucial insights into the disease's impact on brain connectivity and function. Table 1 gives the findings of recent studies on microstructural changes in the CC in Alzheimer's disease.

**Table 1:** Recent image technique studies on microstructural changes in the corpus callosum in Alzheimer's disease FA: fractional anisotropy; MD: mean diffusivity; DTI: diffusion tensor imaging; EOAD: early-onset Alzheimer's disease; PCA: posterior cortical atrophy; IvPPA: logopenic variant primary progressive aphasia; SCD: subjective cognitive decline; aMCI: amnestic mild cognitive impairment

Authors	Study Name	Sample Size	Imaging Technique	Key Findings	Reference
Collij et al.	White matter microstructure disruption in early-stage amyloid pathology	179 subjects	DTI MRI 18F PET imaging	Non-linear relationship observed between regional amyloid burden and white matter microstructure measures in cognitively unimpaired elderly. Initial increase in amyloid corresponded to higher FA and lower diffusivity, followed by lower FA and higher diffusivity with further amyloid increase. Strongest associations found between: Amyloid in the precuneus and FA/MD measures in the body of the corpus callosum Amyloid in the anterior cingulate cortex and axial/radial diffusivity in the genu of the corpus callosum Results suggest initial compensatory mechanisms in white matter with early amyloid accumulation, followed by axonal degeneration patterns in the earliest AD stages	[174]
Mayo et al.	Longitudinal changes in microstructural white matter metrics in Alzheimer's disease	34 individuals with Alzheimer's disease; 33 healthy age-matched controls	DTI	Individuals with Alzheimer's showed decreased FA and increased MD in widespread white matter tracts, including the hippocampal cingulum, at one-year follow-up compared to baseline. Healthy controls also showed decreased FA and increased MD over time, but changes were less extensive and did not include the hippocampal cingulum. Between-group comparisons showed individuals with Alzheimer's disease had lower FA and higher MD compared to healthy controls at baseline and one-year follow-up in multiple regions, including medial temporal areas. Changes in the hippocampal cingulum over one year were specific to the AD group and not seen in healthy controls.	[175]
Caso et al.	White Matter Degeneration in Atypical Alzheimer's Disease	28 patients with EOAD 12 patients with IvPPA 13 patients with PCA Age- and sex-matched healthy control subjects for each group	DTI MRI and voxel-based morphometry	Patients with EOAD, lvPPA, and PCA shared a typical pattern of white matter damage involving the corpus callosum, fornix, and main anterior-posterior pathways. They also shared cortical atrophy in the left temporoparietal regions and precuneus. White matter damage was more severe and widely distributed than cortical atrophy, especially in lvPPA and PCA. EOAD showed additional damage to the genu/splenium of the corpus callosum and parahippocampal tracts bilaterally. The distribution of white matter damage exceeded cortical atrophy and may reflect pathological spread through structural connections. White matter degeneration may be an early marker of Alzheimer's pathology in atypical forms.	[176]
Wang et al.	Abnormal organization of white matter networks in patients with subjective cognitive decline and mild cognitive impairment	36 normal controls 21 SCD patients 33 aMCI patients	DTI	Small-world topology was found in all groups aMCI patients showed increased path length and decreased efficiency compared to normal controls SCD patients had intermediate values between aMCI and normal controls Alterations in regional centrality were found in aMCI patients, particularly in the right precuneus Network metrics correlated with cognitive performance in aMCI patients	[177]
Shu et al.	Disrupted Topologic Efficiency of White Matter Structural Connectome in Individuals with Subjective Cognitive Decline	36 participants with SCD; 51 healthy control participants	DTI	Participants with SCD had less global efficiency and local efficiency in their white matter networks compared to healthy controls Lower regional efficiency was mainly found in bilateral prefrontal regions and left thalamus in SCD participants A disrupted subnetwork consisting of widespread anatomic connections was identified in SCD participants; Similar hub distributions but less connection strength between hub regions were found in SCD participants; Diminished strength of rich club and local connections correlated with impaired memory performance in SCD participants	[178]

As the most prominent WM structure connecting the brain's hemispheres, the CC undergoes notable alterations in Alzheimer's patients. These changes include overall atrophy, thinning of specific regions (particularly the posterior splenium and anterior genu), demyelination of axons, and loss of white matter integrity. Advanced neuroimaging techniques like DTI, MRI, and FA measurements have revealed these subtle structural modifications, which often correlate with cognitive decline.

The microstructural changes in the CC may disrupt interhemispheric communication and impact various cognitive functions, including processing speed and information transfer between hemispheres. These alterations are believed to result from the accumulation of amyloid-β and tau proteins, neuroinflammation, and oxidative stress, key pathological hallmarks of AD. Importantly, these changes could potentially serve as early biomarkers for AD, aiding in diagnosis and differentiation from other forms of dementia. The CC's structural integrity has been shown to correlate with cognitive performance in patients with Alzheimer's disease, suggesting that its degradation may contribute to the progressive cognitive impairment characteristic of the disease.

Understanding these microstructural changes provides valuable insights into the pathophysiology of Alzheimer's and may contribute to the development of new diagnostic and therapeutic strategies. Early detection of CC alterations could facilitate earlier intervention and potentially slow disease progression. Furthermore, tracking these changes over time may offer a means to monitor disease progression and evaluate the efficacy of potential treatments. As research in this area continues, the study of CC microstructure in Alzheimer's disease may play an increasingly important role in both clinical practice and neurodegenerative disease research.

Findings from DTI Studies

DTI studies have significantly advanced our understanding of microstructural changes in the CC in Alzheimer's disease. These studies consistently reveal decreased FA and increased mean diffusivity (MD) in the CC of patients with Alzheimer's disease compared to healthy controls. The changes are particularly pronounced in the splenium (posterior portion) and genu (anterior portion) of the CC, aligning with the overall pattern of atrophy and thinning observed in structural MRI studies.

The sensitivity of DTI allows for the detection of subtle WM alterations that precede visible atrophy on conventional MRI scans. Longitudinal DTI studies have demonstrated that changes in CC integrity can be detected in the early stages of Alzheimer's, potentially even in the preclinical phase. This early detection capability underscores the potential of DTI metrics as sensitive biomarkers for early diagnosis of Alzheimer's disease and progression monitoring.

Importantly, DTI findings in the CC have been shown to correlate strongly with cognitive decline in Alzheimer's patients. Reduced FA and increased MD in specific regions of the CC are associated with impairments in various cognitive domains, including (i) processing speed, often linked to changes in the genu and body of the CC, (ii) executive function, particularly associated with alterations in the genu, (iii) memory, correlated with changes in the splenium, which connects temporal and parietal lobes involved in memory processes, and (iv) global cognitive function, reflected in overall CC integrity. These structure-function relationships highlight the critical role of the CC in maintaining efficient communication between brain hemispheres and supporting various cognitive processes.

Furthermore, DTI studies have shown promise in differentiating Alzheimer's from other forms of dementia, such as frontotemporal dementia and vascular dementia. The pattern and extent of microstructural changes in the CC often differ between these conditions, potentially aiding in differential diagnosis.

The microstructural changes detected by DTI are thought to reflect several underlying pathological processes in Alzheimer's disease: (i) Demyelination of axons, leading to reduced FA and increased MD, (ii) Axonal loss, resulting in decreased directional diffusion of water molecules, (iii) Accumulation of amyloid-β and tau proteins, which may disrupt white matter integrity, and (iv) Neuroinflammation and oxidative stress, contribute to white matter damage.

These processes collectively contribute to the disruption of interhemispheric communication, which may explain some of the cognitive deficits observed in Alzheimer's disease patients.

Recent advances in DTI techniques, such as high angular resolution diffusion imaging (HARDI) and diffusion kurtosis imaging (DKI), are providing even more detailed insights into the microstructural changes of the CC in Alzheimer's. These advanced methods allow for better characterization of complex white matter architectures and may reveal subtle alterations not captured by traditional DTI metrics, as shown in different recent studies in Table 1.

Relationship to Clinical Symptoms

The microstructural alterations in the CC observed in Alzheimer's disease have been found to correlate significantly with various clinical symptoms, providing insight into the relationship between brain structure and function in the disease. This relationship encompasses cognitive, behavioral, and even motor symptoms associated with Alzheimer's progression. In terms of cognitive symptoms, changes in the splenium of the CC, which connects temporal and parietal regions, correlate with episodic memory deficits, likely due to disrupted communication between memory-related brain areas. Alterations in the genu of the CC are associated with declined executive function, including difficulties in planning, decision-making, and cognitive flexibility. Overall CC integrity, particularly in the body region, relates to reduced processing speed, a common early symptom of Alzheimer's disease. Microstructural changes in regions connecting language areas correlate with language deficits, including word-finding difficulties and reduced verbal fluency.

Behavioral symptoms also show connections to CC integrity. Some studies have found associations between CC alterations and apathy levels in Alzheimer's patients, possibly due to disrupted frontal-subcortical circuits. While not consistently observed, some research suggests a link between CC changes and depressive symptoms in Alzheimer's. Changes in interhemispheric connectivity may contribute to behavioral dysregulation, including agitation and aggression in some patients.

Although not typically considered primary Alzheimer's symptoms, changes in CC microstructure have been associated with motor symptoms such as gait disturbances, balance problems, and decline in fine motor skills and coordination, particularly in the later stages of the disease. Overall CC integrity often correlates with global cognitive scores in Alzheimer's patients, reflecting its impact on general cognitive function.

Longitudinal studies have shown that the rate of microstructural changes in the CC can predict the rate of cognitive decline, potentially serving as a prognostic marker. Importantly, subtle changes in CC microstructure may be detectable before overt clinical symptoms appear, suggesting potential as an early biomarker for Alzheimer's disease. These structural alterations also correlate with changes in functional connectivity between brain regions, as measured by fMRI, providing a link between structural and functional brain changes in Alzheimer's.

It's important to note that while these relationships between CC changes and clinical symptoms are significant, they are not perfectly linear or uniform across all patients. The complex nature of AD pathology, involving multiple brain regions and systems, means that CC changes are just one factor contributing to the diverse symptom profile of the disease. Furthermore, the relationship between microstructural changes and symptoms can be bidirectional, with progressive symptoms potentially leading to further degradation of white matter integrity through reduced brain activity and connectivity. Understanding these structure-function relationships not only enhances our comprehension of Alzheimer's pathophysiology but also holds the potential for developing more targeted interventions and better tracking of disease progression in clinical settings., as shown in different recent studies in Table 1 [174-178].

Microstructural changes in the CC in Parkinson's disease

Microstructural changes in the CC are not as extensively studied in Parkinson's disease as they are in Alzheimer's disease, but research has shown that Parkinson's disease does indeed affect this important white matter structure. Parkinson's disease primarily affects the basal ganglia and is characterized by motor symptoms, but it also involves widespread changes throughout the brain, including white matter alterations. The CC, as the largest white matter tract in the brain, is not spared from these changes.

Studies using DTI have revealed microstructural alterations in the CC of Parkinson's disease patients. These changes are generally less pronounced than those seen in Alzheimer's disease but are still significant. The alterations typically include decreased FA and increased MD, indicating compromised white matter integrity. The pattern of changes in the CC in Parkinson's disease appears to be somewhat different from that seen in Alzheimer's disease. While Alzheimer's tends to affect the posterior (splenium) and anterior (genu) portions more severely, in Parkinson's disease, changes are often more diffuse or may preferentially affect different regions.

These microstructural changes have been associated with various clinical features of Parkinson's disease. Cognitive impairment in Parkinson's disease has been linked to CC alterations, particularly in cases of Parkinson's disease with mild cognitive impairment or dementia. The changes may contribute to the disruption of neural networks involved in cognitive processes. Motor symptoms, including gait disturbances and balance problems, have also been associated with CC integrity in some studies. This suggests that interhemispheric communication plays a role in motor control in Parkinson's disease. Non-motor symptoms, such as depression and anxiety, which are common in Parkinson's disease, may also relate to CC changes, though this relationship is less clear and requires further study.

The microstructural changes in the CC may reflect several underlying processes in Parkinson's disease. These include direct effects of alpha-synuclein pathology, the hallmark protein aggregation in Parkinson's disease; secondary effects of neurodegeneration in connected gray matter regions; alterations in neurotransmitter systems, particularly the dopaminergic system; and potential contributions from vascular factors, which are increasingly recognized in Parkinson's disease pathology.

Longitudinal studies have suggested that CC changes may progress throughout Parkinson's disease, potentially correlating with disease progression and worsening of symptoms. Some research has explored the potential of CC DTI measures as biomarkers in Parkinson's disease, particularly for cognitive decline and the development of Parkinson's disease dementia. However, more research is needed to establish their clinical utility.

Comparisons between Parkinson's disease and other neurodegenerative diseases have shown that while CC changes occur in Parkinson's, they are generally less severe than in Alzheimer's disease or some atypical Parkinsonian syndromes like progressive supranuclear palsy. Understanding these microstructural changes in the CC in Parkinson's disease contributes to our overall comprehension of the widespread effects of the disease on brain connectivity. This knowledge may help in developing better diagnostic tools, tracking disease progression, and potentially identifying new therapeutic targets aimed at preserving brain connectivity in Parkinson's disease.

DTI and MRS Findings

Recent neuroimaging studies using DTI and MRS have revealed significant microstructural changes in the CC of individuals with Parkinson's disease. DTI findings consistently show decreased fractional anisotropy and increased mean diffusivity in various parts of the CC, particularly in the genu and splenium, indicating reduced white matter integrity and potential axonal loss or demyelination. These changes are often accompanied by increased radial diffusivity, suggesting myelin degradation. MRS studies, while less common, have reported decreased N-acetyl aspartate/creatine ratios in the CC and surrounding white matter, pointing to neuronal dysfunction or loss. Some studies also note increased choline/creatine ratios and elevated myoinositol levels, which may reflect increased membrane turnover and altered glial function, respectively. These microstructural alterations have been correlated with cognitive impairment, particularly in executive function and processing speed, as well as motor symptom severity in Parkinson's. Collectively, these findings support the view of Parkinson's disease as a widespread neurodegenerative process affecting not only the basal ganglia but also white matter structures like the CC, potentially contributing to interhemispheric disconnection and explaining some of the cognitive and motor symptoms observed in Parkison's disease patients, as shown in different recent studies (Table 2) [179-185].

**Table 2:** Recent image technique studies on microstructural changes in the corpus callosum in Parkinson's disease DTI: diffusion tensor imaging; TBSS: tract-based spatial statistics; PD-MCI: Parkinson's with mild cognitive impairment; FA: fractional anisotropy; H&Y: Hoehn and Yahr; MD: mean diffusivity

Authors	Study Name	Sample Size	Imaging Technique	Key Findings	Reference
Jiang et al.	A novel method for evaluating brain function and microstructural changes in Parkinson's disease	31 Parkinson's disease patients 34 healthy controls	DTI TBSS	Parkinson's patients showed lower FA values in the substantia nigra, putamen, and frontal white matter compared to controls. FA values in the substantia nigra correlated negatively with disease progression in Parkinson's disease. DTI measures in the frontal white matter correlated with depression symptoms in PD patients. TBSS analysis revealed more extensive white matter lesions in Parkinson's patients, including in the corpus callosum, superior longitudinal fasciculus, cingulum bundle, and other regions. FA values in several white matter tracts correlated with cognitive and depressive symptoms in Parkinson's disease.	[179]
Zheng et al.	DTI Correlates of Distinct Cognitive Impairments in Parkinson's Disease	16 patients with Parkinson's disease	DTI	Executive function correlated with DTI measures in frontal white matter tracts, especially the anterior limb of the internal capsule and genu of the corpus callosum. Language and attention performance correlated with DTI measures in frontal regions, with attention also involving widespread areas throughout the brain. Memory impairment mainly involved mean diffusivity alterations within the fornix. No significant correlations were found between visuospatial skills and DTI measures. Distinct patterns of white matter diffusivity were associated with impairments in different cognitive domains in Parkinson's patients.	[180]
Rektor et al.	White matter alterations in Parkinson's disease with normal cognition precede grey matter atrophy.	20 Parkinson's disease patients 21 healthy controls	High-resolution T1-weighted MRI 60 directional diffusion-weighted 3T MRI	Parkinson's patients showed no significant differences from healthy controls in cognitive functioning or brain volumes. Decreased gray matter intensity was found in Parkinson's disease patients' left superior parietal lobe. They showed elevated axial diffusivity in the superior and anterior corona radiata, internal capsule, and external capsule in the left hemisphere compared to controls. Higher mean and radial diffusivity were found in fronto-temporal white matter. White matter impairment in Parkinson's was widespread and preceded significant gray matter alterations, suggesting it may be a sensitive early sign of neurodegeneration.	[181]
Agosta et al.	Mild Cognitive Impairment in Parkinson's Disease Is Associated with a Distributed Pattern of Brain White Matter Damage	43 Parkinson's disease patients (30 PD-MCI, 13 PD-Cu); 33 healthy controls	3D T1-weighted MRI DTI	PD-MCI patients showed decreased fractional anisotropy in multiple white matter tracts compared to healthy controls and Parkinson's Cu patients, including anterior and superior corona radiate; corpus callosum (genu and body); Superior longitudinal fasciculus Inferior fronto-occipital fasciculus; Uncinate fasciculus; No gray matter atrophy was found in PD-MCI patients compared to controls or PD-Cu patients. PD-Cu patients showed no white matter or gray matter differences compared to healthy controls. The white matter damage in PD-MCI was mainly located in frontal brain regions and interhemispheric connections. Cognitive deficits in PD-MCI patients were primarily in executive function and memory domains.	[182]
Deng et al.	Diffusion Tensor Imaging Reveals White Matter Changes Associated with Cognitive Status in Patients with Parkinson's Disease	64 patients with Parkinson's disease: 24 Parkinson's patients with normal cognition (PD-CogNL); 30 Parkinson's patients with mild cognitive impairment (PD-MCI); 10 Parkinson's patients with dementia (PD-D) 21 healthy controls	DTI	PD-D and PD-MCI groups showed significant FA reductions in left frontal and right temporal white matter and bilateral anterior cingulate bundles compared to controls. The PD-D group showed significant FA reductions in a left anterior cingulate bundle and corpus callosum splenium compared to other groups. FA values in some white matter regions negatively correlated with Parkinson's patients' cognitive status. Results suggest white matter damage may be a significant factor in the mechanism of progressive cognitive impairment in Parkinson's. Disease duration increased with worsening cognitive status (PD-CogNL< PD-MCI < PD-D), suggesting progressive deterioration. The study provides evidence that cerebral white matter deterioration may underlie progressive cognitive impairment in Parkinson's disease.	[183]
Lenfeldt et al.	Fractional Anisotropy in the Substantia Nigra in Parkinson's Disease: a Complex Picture	122 patients and 34 controls at baseline Follow-ups at 1, 3, and 5 years	MRI DTI	Increased FA and axial diffusion in the substantia nigra in Parkinson's disease patients compared to controls. Right substantia nigra had higher FA than left in all subareas examined. Hemispheric differences in diffusion measures were observed in the substantia nigra and middle cerebellar peduncle but were not related to disease or symptom lateralization. No progression of diffusion measures over time was observed. Results were consistent across different MRI scanners and follow-up timepoints. Findings challenge previous studies that reported decreased FA in the substantia nigra in Parkinson's disease. Authors suggest increased FA could be explained by inflammation, gliosis, or intrusion of surrounding fibers into the shrinking substantia nigra structure	[184]
Amandola et al.	Longitudinal corpus callosum microstructural decline in early-stage Parkinson's disease in association with akinetic-rigid symptom severity	61 Parkinson's subjects (N=61, aged 45-82, 38 males and 23 females, H&Y ≤ 2)	DTI	Significant FA and MD changes in the corpus callosum over two years, especially in the genu and splenium. Almost all subdivisions of the corpus callosum showed a significant decline in FA over the two-year timeframe Akinetic-rigid severity was negatively associated with callosal FA at baseline and 24 months follow-up, with the most potent effect in anterior corpus callosum; Callosal microstructure alterations in anterior corpus callosum may serve as a biomarker for akinetic-rigid symptomology and disease progression in early Parkinson's.	[185]

Implications for Motor and Cognitive Symptoms

The microstructural changes observed in the CC of Parkinson's disease patients have significant implications for both motor and cognitive symptoms. The CC, as the primary WM tract connecting the two cerebral hemispheres, plays a crucial role in interhemispheric communication and coordination. Alterations in its structure can disrupt this communication, potentially contributing to the asymmetry often seen in Parkinson's disease motor symptoms and the difficulties with bimanual coordination. Cognitively, the CC is involved in various processes including attention, processing speed, and executive function. The observed reductions in white matter integrity, particularly in the genu and splenium, correlate with deficits in these cognitive domains in Parkinson's disease patients. Furthermore, these structural changes may contribute to the development of mild cognitive impairment and dementia in Parkinson's disease by compromising the efficient transfer of information between hemispheres. The relationship between CC alterations and non-motor symptoms such as mood disorders and sleep disturbances in Parkinson's is also an area of ongoing research. Understanding these implications provides valuable insights into the pathophysiology of Parkinson's disease beyond the classic basal ganglia model and may inform future therapeutic strategies aimed at preserving CC integrity or mitigating the effects of its disruption.

Microstructural changes in the CC in Huntington's disease

Huntington's disease, a neurodegenerative disorder characterized by motor, cognitive, and psychiatric symptoms, also exhibits significant microstructural changes in the CC. DTI studies have consistently revealed alterations in WM integrity of the CC in HD patients and even in pre-symptomatic gene carriers. These changes typically include decreased FA and increased MD, particularly in the body and splenium of the CC. Such findings suggest demyelination, axonal damage, or loss of axonal density. Increased radial diffusivity, often observed in Huntington's disease, further supports the hypothesis of myelin degradation. MRS studies, while less numerous, have shown decreased NAA levels in the CC and surrounding WM, indicating neuronal dysfunction or loss. Some studies have also reported increased choline levels, possibly reflecting increased membrane turnover. These microstructural changes in the CC correlate with disease progression, cognitive decline (especially in attention, processing speed, and executive function), and motor symptom severity. Importantly, alterations in the CC have been detected in pre-symptomatic Huntington's disease gene carriers, suggesting that white matter changes may precede the onset of clinical symptoms. This highlights the potential of CC imaging as an early biomarker for Huntington's disease and underscores the widespread nature of the neurodegenerative process in this disease.

The most advanced studies on the microstructural changes in the CC of individuals with Huntington's disease were carried out using diffusional MRI, which facilitated in vivo visualization and detection of the integrity of specific redistribution of the water macromolecules within the area under investigation. Specifically, the method based on the organization and the rate of water diffusion along axonal fiber directions has demonstrated a very versatile application in vivo assays examining the white matter structures of the brain, thus enabling fast, reliable, and noninvasive investigation of brain pathology. Although the results obtained have shown to be very promising in the role of an auxiliary tool in Huntington's disease diagnosis, the imaging biomarkers are still in the early stages of scientific research. Additionally, it is essential to note that the results should be evaluated carefully, and clinical approaches and assessments are constantly necessary for a precise conclusion.

DTI and fMRI Studies

In Huntington's disease, combined DTI and fMRI studies have provided valuable insights into the relationship between structural and functional brain changes. DTI investigations have consistently revealed widespread white matter alterations, with the CC showing significant microstructural damage even in the pre-symptomatic stages of the disease. These changes, characterized by decreased fractional anisotropy and increased mean diffusivity, suggest compromised white matter integrity and potential demyelination. Complementary fMRI studies have demonstrated altered functional connectivity patterns, particularly in motor and cognitive networks. When integrated, these imaging modalities reveal how structural disconnections in the CC and other WM tracts correlate with disrupted functional connectivity between cortical regions. For instance, DTI-detected CC degradation has been associated with reduced interhemispheric functional connectivity in both resting-state and task-based fMRI paradigms. This structural-functional relationship is particularly evident in the motor network, where altered connectivity patterns correlate with the severity of motor symptoms. In the cognitive domain, changes in frontal-striatal structural connectivity detected by DTI have been linked to abnormal activation patterns during executive function tasks in fMRI studies. Importantly, these multimodal imaging findings often correlate with clinical measures of disease progression and cognitive decline, highlighting their potential as biomarkers for tracking Huntington's disease progression and evaluating therapeutic interventions. The integration of DTI and fMRI data in Huntington's disease research thus provides a more comprehensive understanding of how structural neurodegeneration leads to functional brain reorganization, offering crucial insights into the complex pathophysiology of this devastating disorder, as shown in different recent studies (Table 3) [186-191].

**Table 3:** Recent image technique studies on microstructural changes in the corpus callosum in Huntington's disease fMRI: functional magnetic resonance imaging; DWI: diffusion-weighted imaging; CAG: cytosine-adenine-guanine; TRACULA: TRActs Constrained by UnderLying Anatomy; RD: radial diffusivity; AD: axial diffusivity; NODDI: neurite orientation dispersion and density imaging; ODI: Oswestry disability index

Authors	Study Name	Sample Size	Imaging Technique	Key Findings	Reference
Dumas et al.	Reduced functional brain connectivity before and after disease onset in Huntington's disease	20 early Huntington's disease patients (disease stages 1 and 2); 28 premanifest gene carriers 28 healthy controls	Resting-state fMRI	Reduced functional connectivity in premanifest and manifest Huntington's disease compared to controls in: Left middle frontal and pre-central gyrus, Right post-central gyrus, connection with medial visual network. In manifest HD only, reduced connectivity in: Numerous widespread brain regions, Connection with default mode network and executive control network, Differences present even when accounting for atrophy	[186]
Johnson et al.	Dynamics of Cortical Degeneration Over a Decade in Huntington’s Disease	49 Huntington's disease gene carriers and 49 age-matched control participants	Longitudinal structural MRI (3-7 annual scans per participant over 2-6 years)	Widespread cortical volume differences between Huntington's disease and control groups at the time of motor diagnosis; Highest rates of cortical atrophy in occipital and parietal regions during the period surrounding motor diagnosis; CAG repeat length predicted rate of atrophy in occipital, parietal, and striatal regions; Cortical atrophy in specific regions predicted a decline in motor and cognitive performance	[187]
Poudel et al.	Network Spread Determines the Severity of Degeneration and Disconnection in Huntington's disease.	52 total participants (26 symptomatic Huntington's disease patients, 26 age-matched healthy controls)	Structural MRI and DWI	The fifth eigenmode of the healthy brain connectome accurately predicted the cortico-striatal pattern of degeneration in Huntington's disease. Initiating network diffusion from subcortical regions like the accumbens and thalamus generated spatial patterns representing typical neurodegenerative characteristics in Huntington's disease. White matter connections linking nodes with the highest predicted disease spread from the striatum were most vulnerable to disconnection in Huntington's disease. The network diffusion model successfully recapitulated patterns of neural degeneration and disconnection observed in Huntington's disease. Results suggest that trans-neuronal diffusion of mutant huntingtin protein across the brain connectome may explain patterns of gray matter degeneration and white matter disconnection in Huntington's disease.	[188]
Espinoza et al.	Whole-Brain Connectivity Analysis in Huntington's Disease Gene Mutation Carriers	261 total participants (183 Huntington's gene mutation carriers and 78 healthy controls)	Resting-state fMRI	Reduced connectivity within the putamen network as CAG repeat length increases. Decreased functional connectivity between putamen and insula networks associated with increasing CAG repeat length and worse motor/cognitive performance. Decreased functional connectivity between various visual networks as CAG repeat length increases. Increased functional connectivity between calcarine and middle frontal gyrus networks as CAG repeat length increases. When excluding participants with reduced penetrance CAG alleles, additional connectivity alterations were observed, including in default mode network regions.	[189]
Rosas et al.	Complex Spatial and Temporally Defined Myelin and Axonal Degeneration in Huntington's Disease	38 early symptomatic HD patients; 31 pre-manifest HD gene carriers; 37 healthy controls	DWI and tractography using TRACULA	Early changes in RD were found in pre-manifest Huntington's subjects, particularly in tracts like the cingulum, anterior thalamic radiations, superior longitudinal fasciculus, and inferior longitudinal fasciculus. Increases in RD were associated with impaired performance on cognitive tests in domains known to be affected in Huntington's disease. In early symptomatic Huntington's patients, there were widespread increases in AD in multiple white matter tracts. Increases in AD were associated with regional cortical thinning in areas known to atrophy in Huntington's. The findings suggest distinct myelin and axonal degeneration patterns occurring at different stages of Huntington's progression.	[190]
Zhang et al.	In Vivo Characterization of White Matter Pathology in Premanifest Huntington's Disease	38 pre-Huntington's gene carriers and 45 controls	NODDI DTI	Widespread reductions in axonal density (indexed by NDI in pre-Huntington's disease gene carriers compared to controls Localized increases in coherence of axonal organization (indexed by decreased ODI) in white matter surrounding the basal ganglia in pre-Huntington's disease Axonal density reductions in callosal regions correlated with clinical markers of disease progression DTI analysis showed widespread increases in mean diffusivity and localized decreases in FA in pre-Huntington's disease	[191]

Impact on Motor and Cognitive Functions

Microstructural changes in the CC have significant impacts on motor and cognitive functions in Huntington's disease. The CC undergoes notable alterations in Huntington's disease patients. These changes include reduced WM integrity, decreased fiber density, and modifications in myelin structure and axonal organization. Such microstructural alterations disrupt interhemispheric communication, affecting both motor and cognitive processes. In terms of motor function, these changes can impair coordination, contribute to involuntary movements like chorea, and reduce fine motor control and balance. Cognitively, they may lead to decreased processing speed, impaired executive functions, difficulties with attention and working memory, and reduced cognitive flexibility. Neuroimaging studies, particularly using DTI and MRI, have revealed reduced fractional anisotropy and atrophy in the CC of Huntington's disease patients. The extent of these microstructural changes often correlates with disease progression, suggesting that callosal alterations could serve as a biomarker for Huntington's disease severity. The mechanisms behind these changes may involve direct effects of the mutant huntingtin protein on white matter, neuronal loss in connected cortical regions, or secondary effects due to inflammation or metabolic changes. Understanding these impacts has important therapeutic implications, as strategies aimed at preserving callosal integrity could potentially help maintain motor and cognitive functions in Huntington's disease patients. Overall, the microstructural changes in the CC contribute significantly to the complex symptomatology of Huntington's disease, highlighting the importance of white matter pathology in this neurodegenerative disorder.

Microstructural changes in the CC in ALS

Microstructural changes in the CC in ALS have been increasingly recognized as an important aspect of the disease pathology. ALS, primarily known as a motor neuron disease, has been shown to affect various brain regions, including WM structures like the CC. Advanced neuroimaging techniques, particularly DTI, have revealed significant alterations in the microstructure of the CC in ALS patients.

These changes typically manifest as reduced FA and increased MD, indicating a loss of WM integrity. The alterations are often most pronounced in the motor-related areas of the CC, specifically the fibers connecting the primary motor cortices. However, changes have been observed throughout the CC, suggesting a more widespread involvement of interhemispheric connections in ALS.

The microstructural changes in the CC are believed to contribute to both motor and cognitive symptoms in ALS. Motor deficits may be exacerbated by impaired interhemispheric communication, while cognitive changes, particularly in executive function and processing speed, may be partly attributed to these callosal alterations. Importantly, these microstructural changes often precede visible atrophy on conventional MRI, potentially serving as an early biomarker of ALS progression.

The underlying mechanisms of these changes are not fully understood but may involve axonal degeneration, demyelination, or both. Some researchers propose that the CC may be an early site of pathology in ALS, with changes potentially spreading along white matter tracts. The extent of callosal involvement has been correlated with disease severity and progression rate in some studies, further highlighting its clinical relevance.

Understanding these microstructural changes in the CC is crucial for improving our comprehension of ALS pathophysiology, potentially aiding in earlier diagnosis and providing new targets for therapeutic interventions.

DTI and MRS Findings

DTI and MRS have provided valuable insights into the microstructural changes in the CC in ALS. These advanced neuroimaging techniques offer complementary information about structural integrity and metabolic changes in the brain.

DTI findings in ALS consistently show alterations in the CC. The most common observation is a decrease in FA, which indicates reduced WM integrity. This decrease in FA is often accompanied by an increase in MD, RD, and sometimes AD. These changes are typically most pronounced in the motor-related areas of the CC, particularly the fibers connecting the primary motor cortices (corresponding to the middle and posterior body of the CC). However, alterations have been observed throughout the structure, suggesting widespread involvement.

DTI studies have also revealed that these microstructural changes often precede visible atrophy on conventional MRI scans. This makes DTI a potentially valuable tool for early detection of ALS pathology. Some research has shown correlations between the degree of DTI abnormalities in the CC and clinical measures of disease severity and progression.

MRS, on the other hand, provides information about the biochemical composition of brain tissue. In ALS, MRS studies of the CC have shown several significant findings: Decreased NAA levels, which is indicative of neuronal loss or dysfunction. Increased myo-inositol (mI) levels, suggesting glial activation or proliferation. Alterations in choline-containing compounds may reflect changes in membrane turnover or myelin integrity. Changes in glutamate and glutamine levels, potentially indicate alterations in excitatory neurotransmission.

These MRS findings complement the structural information provided by DTI, offering insights into the metabolic and cellular changes occurring in the CC in ALS. Together, these techniques paint a picture of both structural degeneration and altered cellular metabolism in the CC of ALS patients.

The combination of DTI and MRS findings supports the notion that the CC is significantly affected in ALS, with both structural and metabolic changes contributing to the overall pathology. These neuroimaging techniques not only enhance our understanding of the disease process but also hold promise for improving diagnosis, monitoring disease progression, and potentially evaluating the efficacy of future treatments. Table 4 shows different recent studies [192-198].

**Table 4:** Recent image technique studies on microstructural changes in the corpus callosum in ALS DTI: diffusion tensor imaging; FA: fractional anisotropy; ALS: amyotrophic lateral sclerosis; PLS: primary lateral sclerosis; TBSS: tract-based spatial statistics; ALSFRS-R: Revised Amyotrophic Lateral Sclerosis Functional Rating Scale; MD: mean diffusivity; CST: corticospinal tract

Authors	Study Name	Sample Size	Imaging Technique	Key Findings	Reference
Ciccarelli et al.	Investigation of White Matter Pathology in ALS and PLS Using Tract-Based Spatial Statistics	London cohort: 13 ALS patients, 20 controls; Oxford cohort: 13 ALS patients, 6 PLS patients, 21 controls	DTI was analyzed using TBSS	ALS patients showed reduced FA compared to controls in multiple brain regions, including along the corticospinal tract and areas outside the motor network. PLS patients showed lower FA than ALS patients in the body of the corpus callosum and white matter adjacent to the right primary motor cortex. ALS patients had lower FA than PLS patients in white matter adjacent to the right prefrontal cortex. FA in specific brain regions correlated with disease progression rate in both ALS and PLS patients. The study demonstrated that DTI and TBSS can distinguish between ALS and PLS, providing insights into their respective mechanisms of brain damage	[192]
Cirillo et al.	Widespread Microstructural White Matter Involvement in Amyotrophic Lateral Sclerosis: A Whole-Brain DTI Study	19 patients with ALS 20 matched healthy controls	DTI	Reduced FA in the body of corpus callosum in ALS patients, which correlated with clinical upper motor neuron involvement. Reduced FA in white matter tracts from central corpus callosum to primary motor and premotor cortices bilaterally. FA positively correlated with ALSFRS-R scores in white matter underneath the left premotor cortex. Increased radial and mean diffusivity in the genu and splenium of corpus callosum in ALS patients. Correlations between diffusion measures and clinical scores suggest widespread white matter involvement beyond the ALS motor system. Results support the hypothesis of an active bilateral cortical degeneration process with secondary damage to the corpus callosum in ALS.	[193]
Verstraete et al.	Motor Network Degeneration in Amyotrophic Lateral Sclerosis: A Structural and Functional Connectivity Study	12 ALS patients and 12 healthy controls	Structural MRI (cortical thickness measurements) DTI Resting-state fMRI	Cortical thinning was observed in the precentral gyrus (primary motor area) in ALS patients compared to controls. DTI revealed reduced fractional anisotropy (FA) values in ALS patients' corpus callosum and rostral part of the corticospinal tract. Functional connectivity analysis showed preserved overall organization of the motor network in ALS patients, but the level of functional connectedness correlated with disease progression rate. Patients with increased functional connectedness showed a more progressive disease course. The study demonstrated structural motor network deterioration in ALS with preserved functional connectivity measures. The positive correlation between the functional connectedness of the motor network and disease progression rate suggests the spread of disease along functional connections of the motor network.	[194]
Agosta et al.	Assessment of White Matter Tract Damage in Patients with Amyotrophic Lateral Sclerosis: A Diffusion Tensor MR Imaging Tractography Study	24 patients with ALS 20 healthy controls	DTI tractography	Patients with ALS showed significantly decreased FA and significantly increased MD and radial diffusivity of the CST bilaterally compared to healthy controls. Patients with ALS had significantly increased axial diffusivity of the right uncinate fasciculus relative to controls. CST FA values significantly correlated with the rate of disease progression in ALS patients. No significant differences were found in other white matter tracts examined. The study suggests that DTI tractography can detect preferential damage to the CST in mildly disabled ALS patients, which is associated with disease progression rate. It also revealed subtle involvement of the uncinate fasciculus, which may precede behavioral symptoms in ALS.	[195]
Filippini et al.	Corpus callosum involvement is a consistent feature of amyotrophic lateral sclerosis.	24 patients with ALS and 24 healthy controls	DTI and voxel-based morphometry of T1 images	Consistent reduction in fractional anisotropy was found in the corpus callosum of ALS patients, extending to the region of the primary motor cortices. Gray matter reductions were observed in the primary motor, supplementary motor, anterior cingulate, and temporal lobe regions. Callosal involvement was seen even in patients with little clinical upper motor neuron involvement. Findings support the concept of an independent cerebral pathogenic process in ALS. Combining fractional anisotropy, radial diffusivity, and gray matter measures allowed 90% accurate discrimination between ALS patients and controls	[196]
Kasper et al.	Microstructural White Matter Changes Underlying Cognitive and Behavioural Impairment in ALS – An In Vivo Study Using DTI	72 ALS patients and 65 healthy controls	DTI	About 30% of ALS patients showed cognitive impairment. Cognitively impaired ALS patients showed white matter changes in extra-motor regions, mainly frontal areas. Executive and memory performance correlated with fiber tract integrity in large association tracts. Radial diffusivity was the most sensitive DTI marker for cognitive changes	[197]
Iwata et al.	White matter alterations differ in primary lateral sclerosis and amyotrophic lateral sclerosis	19 patients with PLS 18 patients with ALS 19 age-matched controls	DTI Fiber tracking TBSS	Both PLS and ALS patients showed reduced fractional anisotropy and increased MD in the corticospinal tract and callosal motor fibers compared to controls. ALS patients significantly reduced FA in distal portions of the corticospinal tract compared to PLS patients. PLS patients showed more significant changes in subcortical white matter underlying the motor cortex, with reduced volume suggesting tissue loss. Clinical measures of upper motor neuron dysfunction correlated with reduced FA in the corticospinal tract for ALS patients and increased MD and volume loss in the corticospinal tract for PLS patients. Changes in motor fibers of the corpus callosum correlated strongly with changes in corticospinal fibers in patients but not controls. Findings indicate degeneration affects both corticospinal and callosal neurons in the motor cortex in these disorders.	[198]

 *Association with Motor Neuron Degeneration*

The association between microstructural changes in the CC and motor neuron degeneration in ALS is crucial to understanding the disease's pathology and progression [199,200]. This relationship highlights ALS's complex nature as not just a disease of motor neurons but one that affects broader neuronal networks and WM tracts.

Motor neuron degeneration is the hallmark of ALS, primarily affecting the upper motor neurons in the motor cortex and the lower motor neurons in the brainstem and spinal cord [201]. The CC, particularly its motor fibers, connects these upper motor neurons between the two hemispheres. As such, the degeneration of motor neurons is closely linked to the observed changes in the CC.

Research has shown that the microstructural alterations in the CC often correlate with the degree of motor neuron loss. The motor fibers of the CC, which primarily pass through the middle and posterior body, are typically the most affected. This selective vulnerability aligns with the pattern of motor neuron degeneration seen in ALS [202].

Several vital points that elucidate this association are given below.

Propagation of pathology: Some researchers propose that the CC may be a conduit for spreading ALS pathology. The protein aggregates characteristic of ALS, such as TDP-43, may propagate trans-synaptically along the callosal fibers, contributing to the spread of motor neuron degeneration between hemispheres [203].

Wallerian degeneration: As upper motor neurons in the motor cortex degenerate, their axons, which form part of the CC, undergo Wallerian degeneration. This process contributes to the observed microstructural changes and loss of WM integrity in the CC [204].

Cortical hyperexcitability: ALS is associated with cortical hyperexcitability, which may lead to excessive glutamatergic signaling across the CC. This hyperexcitability could contribute to motor neuron death and callosal degeneration [205].

Correlation with clinical features: The extent of callosal involvement often correlates with the severity of motor symptoms and the rate of disease progression. This suggests a close link between callosal integrity and the functional state of motor neurons.

Bilateral spread: The CC's role in interhemispheric communication may explain the often bilateral nature of ALS symptoms, as pathology in one hemisphere can potentially influence the contralateral side via callosal connections.

Extra-motor involvement: While motor fibers are most affected, changes in other parts of the CC align with the known extra-motor involvement in ALS, including cognitive and behavioral symptoms. Understanding this association has important implications for both research and clinical practice. It suggests that monitoring callosal integrity could serve as a biomarker for disease progression and potentially for assessing the efficacy of treatments. Moreover, it underscores the need for therapeutic approaches that target motor neurons and consider preserving white matter integrity and interhemispheric connections.

This association between CC changes and motor neuron degeneration reinforces the view of ALS as a network disorder, emphasizing the importance of considering both gray matter and WM pathology in understanding and treating the disease.

Comparative analysis of CC changes across neurodegenerative diseases

A comparative analysis of CC changes across neurodegenerative diseases reveals similarities and distinct patterns, providing valuable insights into disease-specific pathologies and potential diagnostic markers. This analysis typically encompasses diseases such as Alzheimer's disease, Parkinson's disease, Huntington's disease, and ALS, among others.

CC atrophy is a well-documented feature in Alzheimer's disease, with changes often most pronounced in the posterior regions, particularly the splenium. This pattern aligns with the characteristic posterior-to-anterior progression of cortical atrophy. Microstructural changes, as revealed by DTI, show decreased FA and increased MD, indicating a loss of WM integrity. These changes often correlate with cognitive decline, especially in processing speed and executive function domains [206].

Parkinson's disease, primarily known for its motor symptoms, also shows CC involvement, albeit to a lesser extent than Alzheimer's. In Parkinson's, changes are more subtle and may be more evident in the later stages of the disease. DTI studies have revealed decreased FA in various callosal regions, with some noting more prominent changes in the anterior portions. These alterations are often associated with cognitive impairment in Parkinson's, particularly in executive function and attention [207].

Huntington's disease presents a distinct pattern of CC involvement. Huntington's is characterized by widespread white matter changes, with the CC showing significant atrophy and microstructural alterations. Unlike Alzheimer's, the changes in Huntington's are more diffuse, affecting both anterior and posterior regions of the CC. The extent of callosal involvement in Huntington's often correlates with disease progression and can be observed even in pre-symptomatic gene carriers, suggesting its potential as an early biomarker [208].

In ALS, CC changes are particularly notable in the motor-related areas, specifically the fibers connecting the primary motor cortices. DTI studies in ALS consistently show reduced FA and increased MD in these regions. However, changes are not limited to motor areas, reflecting the multisystem nature of ALS [209]. The pattern of callosal involvement in ALS is often more focal than the widespread changes seen in Huntington's disease.

Comparative analyses have revealed key distinctions in CC involvement across different neurodegenerative diseases. Regional specificity is one such distinction, where Alzheimer's disease predominantly affects the posterior regions, while Parkinson's disease may show more anterior involvement. Huntington's disease and ALS tend to have more widespread and motor-specific changes, respectively. The correlation with symptoms varies across diseases, with callosal changes correlating with cognitive symptoms in Alzheimer's and Parkinson's, both motor and cognitive symptoms in Huntington's disease, and primarily motor symptoms in ALS.

The timing of callosal changes also differs among these diseases. In Huntington's disease, alterations appear early in the disease course and can precede clinical symptoms. In Alzheimer's disease, callosal changes often parallel cortical atrophy, whereas in Parkinson's disease, they may become more evident in advanced stages. The extent of involvement is another distinguishing factor, with Huntington's disease typically showing the most extensive callosal changes, while Parkinson's disease often has the most subtle alterations.

CC changes show promise as biomarkers across all these diseases, although their utility varies. They may be particularly valuable in Huntington's disease for early detection and in ALS for monitoring disease progression. This comparative analysis highlights the importance of the CC in neurodegenerative processes and underscores its potential as a structure of interest for differential diagnosis and monitoring disease progression. It also emphasizes the need for disease-specific approaches in interpreting callosal changes within the context of each neurodegenerative disorder.

The distinct patterns of CC involvement across these diseases reflect each disorder's unique pathological processes, providing a window into the differential vulnerabilities of brain networks in neurodegeneration. This understanding enhances our ability to develop targeted diagnostic and therapeutic strategies tailored to the specific characteristics of each neurodegenerative disease.

Common patterns and unique features

When examining CC changes across neurodegenerative diseases, we can observe common patterns and unique features. This comparison provides valuable insights into these disorders' shared and distinctive aspects, enhancing our understanding of their pathophysiology and potential diagnostic markers. Across neurodegenerative diseases, the CC typically exhibits a consistent pattern of decreased WM integrity, often evidenced by reduced FA and increased MD in DTI studies [210]. The extent of CC changes in most neurodegenerative diseases correlates with disease severity and progression, suggesting that callosal alterations could serve as a potential biomarker for disease advancement. These changes are frequently associated with impairments in interhemispheric communication, affecting both motor and cognitive functions. While the specific patterns may differ, most neurodegenerative diseases affect multiple regions of the CC rather than being limited to a single area. In several diseases, mainly Huntington's and Alzheimer's, CC alterations can be detected before the onset of clinical symptoms, highlighting their potential for early diagnosis.

Unique features of each disease provide additional insights. In Alzheimer's disease, there is predominant posterior (splenium) involvement, with a strong correlation with memory and cognitive decline, aligning with the posterior-to-anterior progression of cortical atrophy [211]. Parkinson's disease shows more subtle changes compared to other neurodegenerative diseases, with possible anterior predominance in some studies, and changes are more evident in later stages and in patients with cognitive impairment [212]. Huntington's disease involves widespread and severe CC involvement, with changes detectable in presymptomatic gene carriers, affecting both anterior and posterior regions diffusely [213]. In ALS, there is prominent involvement of motor-related areas of the CC, with changes often preceding visible atrophy on conventional MRI and a strong correlation between motor symptoms and upper motor neuron involvement [214]. Multiple sclerosis exhibits a distinct pattern of focal lesions in the CC, often visible on conventional MRI and advanced imaging techniques, with changes that can be dynamic, reflecting periods of acute inflammation and chronic degeneration [215].

These common patterns and unique features reflect the underlying pathological processes specific to each disease. For instance, the posterior predominance in Alzheimer's aligns with the pattern of cortical atrophy, while the motor area involvement in ALS reflects the primary degeneration of motor neurons. The widespread changes in Huntington's are consistent with the global nature of the genetic defect in this disease. Understanding these similarities and differences is crucial for several reasons. The unique patterns can help distinguish between different neurodegenerative diseases, especially in early or atypical presentations, aiding in differential diagnosis. The common correlation with disease progression suggests that CC changes could be used to monitor disease advancement and potentially treatment efficacy. The distinct patterns provide clues about the underlying disease mechanisms and the vulnerability of different brain networks in each disorder, offering pathophysiological insights. Both the standard and unique features could be leveraged to develop imaging biomarkers for early diagnosis, prognosis, and treatment response monitoring. Understanding the specific nature of CC involvement in each disease could lead to targeted therapeutic approaches to preserve white matter integrity and interhemispheric communication.

While neurodegenerative diseases share some common patterns of CC involvement, the unique features of each disorder provide valuable diagnostic and pathophysiological insights. This comparative perspective enhances our understanding of these diseases and allows for improved diagnosis, monitoring, and potential treatment strategies.

Potential biomarkers and therapeutic targets

The investigation of CC changes in neurodegenerative diseases has revealed significant potential for developing biomarkers and identifying therapeutic targets. This dual approach offers promising avenues for improving diagnosis, monitoring disease progression, and developing new treatment strategies. Structural imaging biomarkers, such as patterns of CC atrophy and regional volume loss, can serve as indicators; for instance, posterior callosal atrophy in Alzheimer's disease or motor area atrophy in ALS can be quantified for diagnostic purposes. The rate of callosal atrophy over time could indicate disease progression and potentially treatment efficacy. DTI metrics like FA and MD changes in specific callosal regions also show promise as sensitive biomarkers. These metrics could detect early microstructural changes before visible atrophy, enabling earlier diagnosis and intervention. MRS markers could be used to identify metabolic changes in the CC, such as decreased NAA levels or increased myoinositol, serving as biomarkers of neuronal loss and glial activation, respectively. Integrating structural, diffusion, and spectroscopic measures could provide more robust and specific biomarkers for different neurodegenerative diseases. Tracking changes in these biomarkers over time could offer insights into disease progression and potentially predict clinical outcomes.

In terms of therapeutic targets, several approaches are under exploration. Therapies to maintain myelin integrity and axonal health in the CC could be developed, including approaches to support oligodendrocyte function or reduce oxidative stress. Given the role of neuroinflammation in many neurodegenerative diseases, targeting inflammatory processes in the CC could be a valuable therapeutic approach. In diseases characterized by protein aggregation, such as tau in Alzheimer's disease or TDP-43 in ALS, therapies could target the spread of these pathological proteins through callosal fibers [216]. Enhancing the delivery or production of neurotrophic factors could support callosal axon health and potentially promote regeneration. Therapies aimed at normalizing neuronal excitability could be beneficial in diseases like ALS, where cortical hyperexcitability may contribute to callosal degeneration. Improving or maintaining interhemispheric signal transmission through therapies, potentially via neuromodulation techniques, could help mitigate functional deficits. Approaches to modulate glial cell function, particularly astrocytes, and microglia, could help maintain a supportive environment for axons in the CC. Gene therapy approaches that deliver therapeutic genes to callosal neurons or surrounding glial cells could potentially slow or halt the progression of genetic disorders like Huntington's disease. Additionally, therapies aimed at enhancing neuroplasticity and promoting the formation of new interhemispheric connections could help compensate for callosal degeneration.

Challenges and future directions

The study of CC changes in neurodegenerative diseases has made significant strides, but several challenges remain, and numerous future directions are emerging. Addressing these challenges and pursuing new avenues of research will be crucial for advancing our understanding and improving patient care.

Challenges

Heterogeneity of diseases: Neurodegenerative diseases are highly heterogeneous, with varying clinical presentations and progression rates. This variability makes it difficult to establish universal biomarkers or therapeutic approaches based on CC changes.

Technical limitations: While current imaging techniques are advanced, they still have limitations in resolution and specificity. Improving the sensitivity and resolution of neuroimaging methods is crucial for detecting subtle changes in the CC.

Longitudinal studies: More long-term studies are needed to track CC changes from preclinical stages through disease progression. Such studies are resource-intensive and challenging to conduct.

Integration of multiple biomarkers: While CC changes are promising, they must be integrated with other biomarkers (e.g., genetic, biochemical) for a more comprehensive understanding of disease processes.

Translation to clinical practice: Translating research findings into practical diagnostic tools and therapeutic interventions remains a significant challenge. This includes standardizing imaging protocols and developing clinically feasible assessment methods.

Understanding causality: Determining whether CC changes are causative factors in disease progression or secondary effects of overall brain degeneration is complex and requires further investigation.

Individual variability: The high degree of inter-individual variability in CC structure and function complicates the development of standardized diagnostic criteria and therapeutic approaches.

Future Directions

Advanced imaging techniques: Developing and refining new imaging modalities, such as ultra-high field MRI or advanced diffusion imaging techniques, to provide more detailed insights into callosal microstructure.

Artificial intelligence (AI) and machine learning: Leveraging AI and machine learning algorithms to analyze complex imaging data, potentially identifying subtle patterns of CC changes that may not be apparent through conventional analysis.

Multimodal biomarker integration: Combining CC imaging biomarkers with other modalities such as genetic profiling, blood-based biomarkers, and cognitive assessments for a more comprehensive disease characterization.

Personalized medicine approaches: Developing individualized diagnostic and treatment strategies based on specific patterns of CC changes and other patient-specific factors.

Targeted drug delivery: Exploring methods for targeted drug delivery to the CC, potentially using nanotechnology or other advanced delivery systems.

Neuromodulation techniques: We are investigating noninvasive neuromodulation techniques (e.g., transcranial magnetic stimulation) to potentially enhance CC function or slow degeneration.

Cellular and molecular studies: Conducting more detailed cellular and molecular studies of the CC in neurodegenerative diseases to understand the underlying mechanisms of degeneration and potential for regeneration.

Comparative studies across species: Expanding comparative studies across different species to better understand the evolutionary aspects of CC function and its vulnerability in neurodegenerative diseases.

Focus on presymptomatic stages: Research efforts are increasing on the presymptomatic stages of neurodegenerative diseases to identify early CC changes that might predict disease onset or progression. 

Therapeutic interventions: Developing and testing interventions that preserve CC integrity, such as myelin-protective therapies or axonal growth-promoting factors.

Functional connectivity studies: Expanding research on how CC structural changes relate to alterations in functional connectivity between brain hemispheres.

Environmental factors: Investigating the impact of environmental factors on CC integrity in the context of neurodegenerative diseases.

Longitudinal population studies: Large-scale, long-term population studies track CC changes across the lifespan and examine various risk factors for neurodegenerative diseases.

By addressing these challenges and pursuing these future directions, researchers can enhance our understanding of the role of the CC in neurodegenerative diseases. This could lead to improved early diagnosis, more accurate prognosis, and the development of novel therapeutic strategies. The ultimate goal is to translate these advancements into better patient care and outcomes for those affected by neurodegenerative disorders.

## Conclusions

This review highlights the significant role of CC alterations in various neurodegenerative diseases, including Alzheimer's disease, Parkinson's disease, Huntington's disease, and ALS. Advanced neuroimaging techniques, particularly diffusion tensor imaging and magnetic resonance spectroscopy, have revealed distinct patterns of microstructural changes specific to each disorder, offering valuable insights into disease pathophysiology and progression. These callosal alterations not only reflect the underlying neurodegenerative processes but also contribute to the complex symptomatology observed in these conditions, affecting both motor and cognitive functions.

The CC's involvement across these diseases underscores its potential as a biomarker for early diagnosis, disease monitoring, and treatment efficacy assessment. As research in this field progresses, integrating CC imaging with other biomarkers and expanding our understanding of its role in neurodegeneration may pave the way for more targeted therapeutic interventions and improved patient care. Future studies addressing the challenges of disease heterogeneity, imaging standardization, and translation to clinical practice will be crucial in fully realizing the potential of CC analysis in the management of neurodegenerative diseases.
